# From Hume to Wuhan: An Epistemological Journey on the Problem of Induction in COVID-19 Machine Learning Models and its Impact Upon Medical Research

**DOI:** 10.1109/ACCESS.2021.3095222

**Published:** 2021-07-06

**Authors:** Carlos Vega

**Affiliations:** Luxembourg Centre for Systems Biomedicine, Bioinformatics Core GroupUniversité du Luxembourg 81872 4365 Esch-sur-Alzette Luxembourg

**Keywords:** Biomedical imaging, machine learning, philosophical considerations, computational systems biology, X-rays

## Abstract

Advances in computer science have transformed the way artificial intelligence is employed in academia, with Machine Learning (ML) methods easily available to researchers from diverse areas thanks to intuitive frameworks that yield extraordinary results. Notwithstanding, current trends in the mainstream ML community tend to emphasise *wins* over knowledge, putting the scientific method aside, and focusing on maximising metrics of interest. Methodological flaws lead to poor justification of method choice, which in turn leads to disregard the limitations of the methods employed, ultimately putting at risk the translation of solutions into real-world clinical settings. This work exemplifies the impact of the problem of induction in medical research, studying the methodological issues of recent solutions for computer-aided diagnosis of COVID-19 from chest X-Ray images.

## I. Introduction

To respond to the overwhelming needs arising from the COVID-19 pandemic, a lot of efforts have been put into building computer-aided diagnosis solutions using machine learning methods, hoping to speed up the early detection of this novel coronavirus. This work aims to raise awareness of the risks of building models for computer-aided diagnosis without the appropriate methodologies to justify the methods employed. In particular, the countless solutions aimed at computer-aided diagnosis of COVID-19 from chest radiographs (CXR) images that are not suited for clinical use (see Table 1). The recent literature about COVID-19 solutions already brought to light transversal issues that extend beyond the problem tackled in such works, questioning the methods employed [1] and highlighting the poor quality of the datasets [2]. Conversely, this work focuses on the methodological flaws derived from the lack of domain knowledge that affect how the problems are formulated in the first place and how methods are justified.

**TABLE 1:** Summary of Conventional ML Solutions Employing CXR for Computer-Aided Diagnosis of COVID-19

Binary	Multi-class	Both
Minaee et al. [27] Zhang et al. [29] Ismael and Şengür [30] Ahishali et al. [31] Abraham and Nair [32]	Khan et al. [28] Apostolopoulos and Mpesiana [33] Abbas et al. [34] Mangal et al. [25] Yoo et al. [35] Wang et al. [26] Pereira et al. [36]	Ozturk et al. [37] Toraman et al. [38] Karakanis and Leontidis [39] Narin et al. [40]

It is directed at a general audience, regardless of their field of expertise, and tackles the topic horizontally, from different angles such as Machine Learning (ML), epistemology, probability and pathology. Consequently, this work does not intend to be technically exhaustive in regards to the aforementioned fields of science. More generally, the issues described in this paper concern the scientific method, with knowledge as the ultimate goal of science. In this sense, current mainstream ML community shows a trend to emphasise *wins* over knowledge, specially within the *challenge* culture [3]. Such competitions have an increasing scientific impact in areas such as biomedical image analysis, but lack enough quality control for the translation of solutions into clinical practice [4], raising ethical and legal concerns regarding the diffusion of responsibility and liability [5]–[7].

The lessons and critic of this work can be extended to similar problems and challenges of translational medicine beyond the scope of COVID-19. The most direct consequence of the issues addressed in this paper relates to the poor transferability of computer-aided diagnosis solutions into hospitals and less of rigorous review and approval (e.g. by regulators), in which research institutions have invested their efforts, budget and resources with questionable results [8]–[10].

The rest of the paper is structured as follows: The remainder of this section provides some context on the problem of induction, an example of its limitations, and how they affect our case at issue. Section II describes how conventional ML solutions suffer from previously described problems. Finally, Section III discusses the impact on medical research.

### A. Context

In 1739, still under the shadow of the bubonic plague in Europe, David Hume publishes *A Treatise of Human Nature*, presumably without knowing that his work would not only continue to be debated more than 200 years later, but also still remarkably relevant in the technological advances of our time. In *the problem of induction* Hume argues that we cannot make a causal inference just by *a priori* means, and poses the question of how we can conclude from the observed to the unobserved. For instance, we can certainly assert that every morning the sun rises approximately in the east thanks to our everyday observations, but that is not enough to explain *why* it happens in such a way; for that we would need a bit of domain knowledge [11, Ch. VI].

In ancient Rome, andabatas used to fight blindfolded in the arena, unaware of the outer beasts. Regardless of their training they were doomed to fail. Similarly, we are often told ML models are black boxes whose inside cannot be inspected, but equally dangerous is the fact that models cannot see what surrounds them. Models best hope is to expect their target data resembles that from their training period. In this sense, ML models can be regarded as inductive machines performing inductive inferences based on previous observations. The key performance indicator (KPI) of a ML model is the generalisation performance, measured by how well it will generalise and perform on novel data [12, Ch. 9.4, p. 100]. For the ML model to perform well on novel data, is often assumed that novel data will resemble past data. Hume refers to this assumption as the Principle of Uniformity of Nature.

*If reason determined us, it would proceed upon that principle, that instances, of which we have had no experience, must resemble those, of which we have had experience, and that the course of nature continues always uniformly the same.*

(A Treatise Upon Human Nature [13] T. 1.3.6.4)

But the course of nature does not *necessarily* continue always uniformly the same; especially when *nature* does not refer to the whole universe, but the particular realm where a ML model is employed. Before proceeding with an example of this issue, (see § I-B), we have to tackle two other issues.

Suppose we aim to predict whether the next president of the United States of America will be a woman or not. If we rely solely on the gender of previous presidents, by induction we will predict a zero chance. But by understanding how a person becomes a presidential candidate, and how previously became a candidate for their party, we can take into account the network of people involved in the process and recalculate our forecast with higher precision. In this case the rules are clearly defined in the law. Pouring these bits of domain knowledge into our model will show that chances are increasing over time. Encoding the rules behind the data heavily increased the robustness and precision of our model. Thanks to these rules our inference became deductive rather than inductive, since the conclusion necessarily follows from the premises; and as long as the premises are true the conclusion will also be true.

We can identify two issues in the first approach of our example: First, partial data can misrepresent the underlying phenomena that shapes the data, producing a model that does not resemble the real world. This is especially notable in the case of bias and confounders which are further aggravated by the lack of domain knowledge in designing the solutions. The second issue relates to induction. Contrary to deduction, where the truth of the premises guarantees the truth of the conclusion, inductive inferences are *ampliative*— since whose conclusions go beyond what is contained in their premises — and their conclusions could be totally wrong even if infinitely many examples confirm them [14]. This *ampliative* factor has also an amplifying effect over the partial data from which we infer a conclusion. In this case, considering only the final results of the elections amplified the bias derived from a partial collection of the data, reducing the chances of women being predicted as president to zero. We will later discuss other amplifying effects derived from induction such as the abuse of the outcome space (see § II-A).

These two issues put at risk the transferability of the solutions to real-world clinical settings. There will not be translational solutions without embedding medical knowledge into their development process. The challenges to transfer a solution to the clinical environment condition how data must be collected and curated. These steps are often skipped from the process, with researchers rushing to develop solutions with whatever data is available, regardless of their quality, coverage or suitability. Translational medicine requires robust and adaptable solutions developed and designed upon methodologies that allow for the aforementioned qualities.

### B. The Case at Issue

Now consider a ML model trained to predict different respiratory diseases (e.g. [15]–[17]) such as tuberculosis (TB), asthma, pneumonia, etc. Then, in December 2019, Dr. Zhang Jixian started to treat a pneumonia case of unknown cause [18]. What kind of inference and data led scientists to think it was caused by a new virus? (see § II-B). A chest computer tomography (CT) showed unusual changes in the lungs which were different from any known viral pneumonia. Later on, genetic sequencing related the new virus to coronaviruses that circulate in bats, including SARS [19]. In February 2020 the virus causing COVID-19 (Coronavirus disease 2019) was named SARS-CoV-2 (Severe acute respiratory syndrome coronavirus 2).

The aforementioned ML models, created before the pandemic, were trained for a particular subset of the vast hypothesis space for the prediction of respiratory human diseases. Naturally, they were not trained for this novel disease, so the now outdated biases and weights of the model neurons cannot guess this new disease. Just as doctors who were not aware of this novel disease could not provide an accurate diagnosis prior to its discovery, misidentifying the patient symptoms with those from common cold [20] or measles [21]. In fact, the pathological^1^ findings shown in CXR and CT scans are actually non specific, and overlap with other viral infections (e.g. H1N1, SARS, MERS) [23]. Further on, we will see how this problem affects conventional classifiers.^1^The 2nd word sense of pathology is used in this paper to refer to the physical manifestations of a disease in tissues and organs. Literature often refers to them as abnormalities, signs, patterns, lesions, etc. [22]

## II. Conventional Machine Learning Solutions

Right after the first outbreaks, information and data about COVID-19 flooded the internet, including several datasets and repositories with CXR and CT images of COVID-19 patients [24]. Researchers rushed to develop models using data from such repositories [25]–[27], claiming that such models could be a “very helpful tool for clinical practitioners and radiologists to aid them in diagnosis” [28]. Exhaustive systematic reviews of models and datasets for COVID-19 detection from CXR and CT scans can be found in [1], [2], respectively, addressing the poor suitability of such models for clinical use. Most of these solutions are based on conventional classifiers such as binary and multi-class classification methods. Table 1 presents some examples from the literature. Below we discuss the limitations of this type of models.

### A. Binary and Multi-Class Classification

The problem of classifying a given input into one class out of several classes needs some compromises but also requires some conditions. Typically, these classifiers return a probability for each class, and the most probable class is chosen as prediction. Of course, the sum of these probabilities must be one (see Eq. 1), but reducing the outcome space from the vast sample space to a limited event space of a couple of classes artificially increases the probability of the chosen classes. No matter how many classes are considered, the probability sum of such classes will be one in the training and test sets, but nothing ensures that such events follow the same probability in the real world where more classes exist.

This abuse of the outcome space entails dangerous consequences if a classifier is deployed in a clinical environment different from the train and test set. Even if similar, a real clinical environment is prone to change, a new disease may appear and the model is forced to choose between the set of classes it was trained to classify. Simply put, a model of this kind cannot say *I don’t know*, and therefore reducing its outcome space necessarily increases the probability of the rest of the classes to be mispredicted by the model.

\begin{equation*} P\left ({\Omega }\right)\; =\; 1\tag{1}\end{equation*}

Kolmogorov’s second axiom. The probability of the entire outcome space is 1.

**FIGURE 1.**
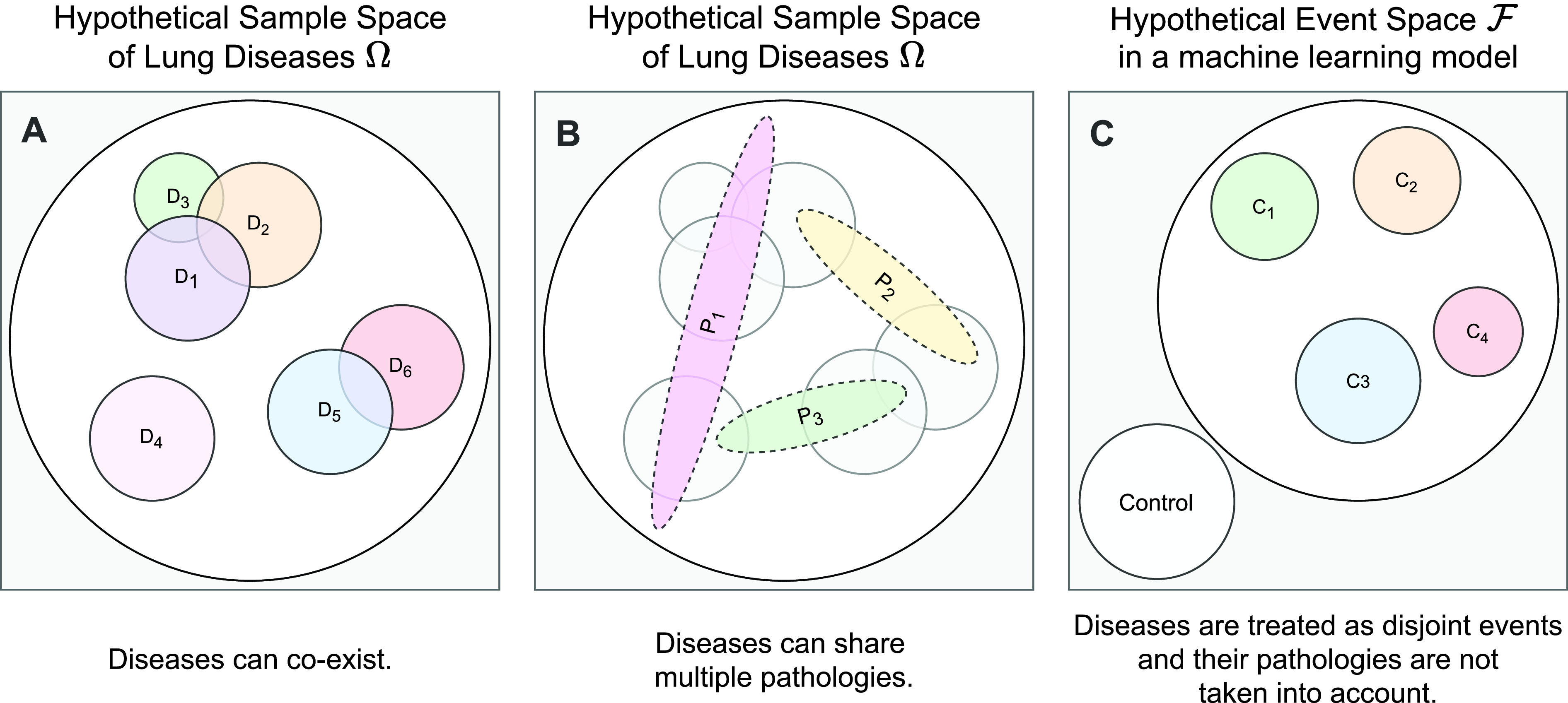
Conventional models often oversimplify the event space and ignore disease etiologies.

Several solutions for diagnosis of COVID-19 [33], [40] limit their outcome space to a couple of diseases, disregarding other many possible lung diseases such as tuberculosis or asthma. Furthermore, these solutions assume that the different lung diseases of the model outcome space are mutually exclusive events (see Figure 1), while in fact, many lung diseases can co-exist (e.g. COVID-19 and TB [41]–[43]) and often share common abnormalities (e.g. consolidations, opacities). Diseases are not found in nature as entities *per se*; they refer to a definable deviation from a normal phenotype evident via symptoms and/or signs. The different sets of symptoms, pathologies and signs are grouped into diseases, and likewise diseases are grouped into categories, all of them organised into disease taxonomies (e.g. International Classification of Diseases). Thus, one disease can have more than one etiology, and one etiology can lead to more than one disease [44]. Lung diseases in particular produce a spectrum of lung pathologies that evolves over time and whose diagnosis requires a combination of tests (e.g. radiology, pulmonary function, blood, sputum, etc). Importantly, some diseases of the respiratory system (e.g. pulmonary vasculature) can be associated with a normal CXR [45]–[47] requiring CT scans and further tests to clarify the diagnosis and prognosis of such cases. Moreover, ML methods do not necessarily capture model predictive uncertainty, adding another source of risk for their predictions in real world settings [48]. Bayesian methods can help quantifying uncertainty caused by the model structure or the use of limited samples (i.e. epistemic uncertainty) [49].

In this light, binary and multiclass solutions for the prediction of lung diseases based on CXR images cannot be translated to the clinical practice, regardless of their accuracy results, since they are based on unrealistic assumptions about the nature of what they predict, and employ predictors not suited for the problem at hand.

### B. Diagnosis and Monotonicity

The issue previously described is also related to the concept of monotonicity. In its epistemic sense, monotonicity expresses the fact that adding more premises to an argument allows you to derive all the same conclusions as you could with fewer [50]. Specifically, under monotonic reasoning, if a conclusion $p$ follows from a set of premises $A$, (denoted as $A \vdash p$), adding another set of premises $B$ doesn’t alter the conclusion (i.e. $A \land B \vdash p$ also holds) [51], [52]. As stated by Pearl: “The problem of monotonic logic lies not in the hardness of its truth values, but rather in its inability to process context-dependent information” [53, Ch. 1.5, p. 24]. Therefore, reasoning is non-monotonic when a conclusion supported by a set of premises can be retracted in the light of new information. Or in other words, we can infer certain conclusions from a subset of a set $S$ of premises which cannot be inferred from $S$ as a whole. Medical diagnosis fits very well under such definition. In the case at issue of this work, the presence of more abnormalities could imply a different etiology, and thus a different disease.

Section I-B left open the question about what kind of inference led scientists to conclude that the abnormal cases of pneumonia treated by Dr. Zhang were caused by a novel coronavirus. Defeasible reasoning deals with tentative relationships between premises and conclusions, which can be *defeated* by additional information, allowing for the retraction of inferences. For instance, while we may infer that Tweety flies based on the information that Tweety is a bird and the domain knowledge that birds generally fly, we can retract this inference when we learn that Tweety is a penguin. Tweety is indeed a bird but it cannot fly.

Defeasible reasoning is also not exempt from limitations, requiring from causal information to properly derive conclusions under certain scenarios [54], [55]. Consider, for example, this problem of Pearl: if the sprinkler is on, then normally the sidewalk is wet, and, if the sidewalk is wet, then normally it is raining. However, we should not infer that it is raining from the fact that the sprinkler is on [53, Ch. 1.5, p. 24].

**FIGURE 2.**
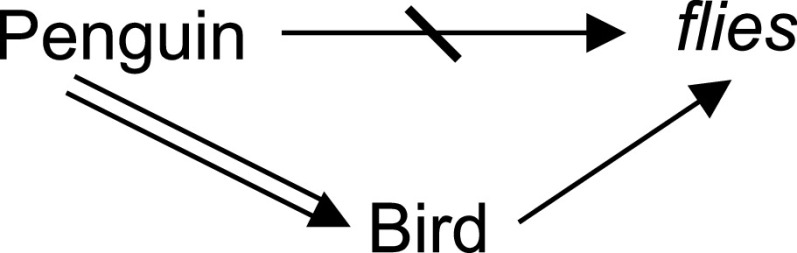
Double arrows indicate non-defeasible inferences (hard fact), single arrows depict defeasible inferences, and strikethrough arrows denote a negation. It can be read as: Penguins are birds (no exceptions); Birds usually fly; and Penguins usually don’t fly.

Conflicts may arise between hard facts and defeasible conclusions. For instance, both arguments in Figure 2 $Penguin \Rightarrow Bird \rightarrow flies$ and $Penguin \rightarrow \neg flies$ finish with a defeasible inference. The transitivity rule $(a~\rightarrow ~b, b~\rightarrow ~c) \Rightarrow a \rightarrow c$ cannot be applied to the first argument. In this case, according to their specificity we can give priority to the argument with more a specific antecedent but is not always as trivial, and complex conflicts can remain unresolved.

During the last decades, non-monotonic logic, defeasible reasoning and causal reasoning have been investigated in Artificial Inteligence (AI) regarding the medical fields [56], [57]. However, methodologies and methods associated with such concepts have not been incorporated into the mainstream ML community yet. Challenges promoting *wins* over knowledge do not help incorporating more complex methods into the mainstream tools, limiting the success assessment of the solutions to the KPIs of interest.

### C. Circumventing the Issues

**FIGURE 3.**
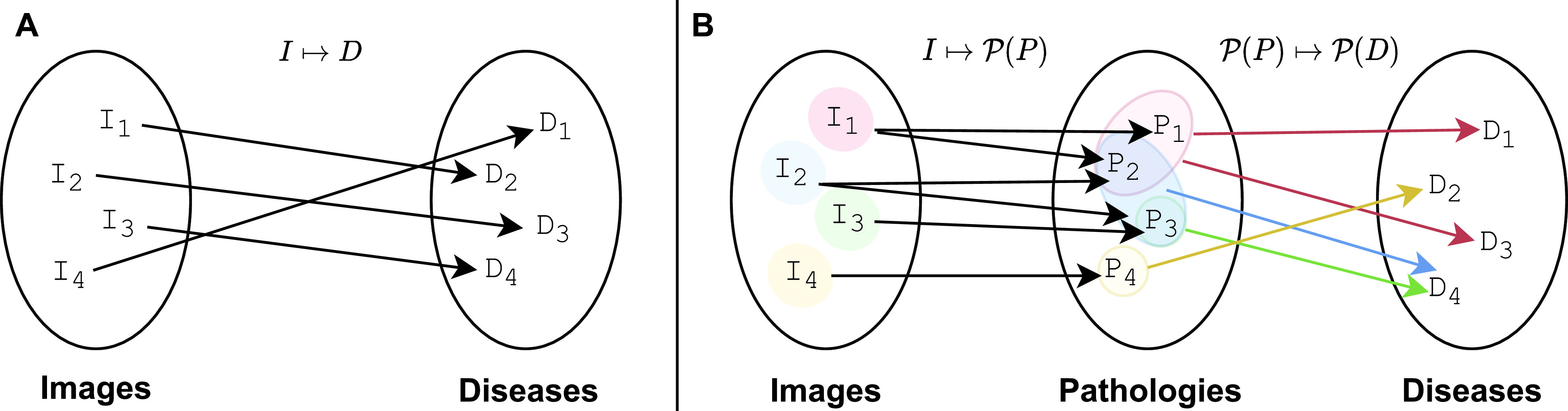
A. Conventional models make use of a single valued mapping, $I \mapsto D$. B. An example of an alternative method employs multivariate and multivalued functions for a more representative mapping. $\mathcal {P}(P)$ and $\mathcal {P}(D)$ denote the respective powersets of the set of pathologies $P$ and set of diseases $D$.

During Section II-A two main issues were identified in the conventional solutions from the literature. First, mapping an image to a single disease (denoted as $I \mapsto D$) is partial and imprecise considering that diseases can co-exist and are not mutually exclusive events. Second, diseases can share pathologies and the pathologies from a particular disease can manifest differently and evolve over time. To workaround these issues, we can follow a process similar to the radiologist diagnosis, which should at least involve two steps. First, deriving a set of pathologies from a given image, $I \mapsto \mathcal {P}(P)$ where $\mathcal {P}(P)$ denotes the powerset^2^ of the pathologies set $P$, i.e. the set of all subsets of $P$, including the empty set and $P$ itself. Second, once the pathologies have been derived from a given image $I$, they can be mapped to diseases from the set of diseases $D$, $\mathcal {P}(P) \mapsto \mathcal {P}(D)$. Figure 3 depicts a comparison of the previously described processes.^2^Also written as $2^{P}$ in set theory.

Note that the second step $\mathcal {P}(P) \mapsto \mathcal {P}(D)$ could be enriched with extra information such as preconditions, tests results etc. for an increased precision. Moreover, radiologists often refer to the set of abnormalities found in the images as patterns, in this vein, each disease could alternatively be provided with a corresponding function as denoted in Equation 2, providing a *matching* value for a set of pathologies taken as argument.

\begin{equation*} \forall d \in \mathcal {F}, f_{D}: \mathcal {P}(P) \mapsto m_{d}\tag{2}\end{equation*}

Let $\mathcal {F}$ be the event space of diseases ${d_{1}, d_{2}, \ldots, d_{n}}$.

To dig a little more into the example, it could be possible to define a dedicated function for each disease $D$ in a form similar to the example below (see eq. 3). This function would take into account the different abnormalities detected in the CXR and express the combination of pathologies that matches the disease definition

\begin{equation*} \forall d \in \mathcal {F}: f_{d} = \sum _{p \in d} \#_{p} \triangle _{p} w_{p} \tag{3}\end{equation*}

Let $\Omega $ be the sample space of all pathologies ${p_{1}, \ldots, p_{n}}$ and $\mathcal {F}$ the event space of diseases ${d_{1}, \ldots, d_{n} }$ with $\#_{p}$ as the number of occurrences of a given pathology $p$; $\triangle _{p}$ being the total area of the pathology on all its occurrences; and $w_{p}$ the pathology relevance.

Likewise, example Equation 3 could be (and certainly should be) enriched with the additional multi-modal information derived from other tests (e.g. blood, sputum, etc). This extra information can have a different degree of relevance in the diagnosis, and even override the rest of the factors in the equation, requiring more complex functions than the above examples. Such equations are precisely where the pathology knowledge should be embedded, encoding the relevant parameters in the form of a formula together with additional information relevant for the diagnosis. For instance, a culture positive of *Mycobacterium tuberculosis* can suffice to diagnose TB, even with a normal CXR. On the other hand, a negative culture can cancel the rest of the parameters derived from an abnormal CXR, at least for this particular disease. [58].

All previous examples are not final but just indicative from a different method to detect diseases without incurring in the issues described in Section II-A. These methods are not novel and are often used in different areas. For instance, using a ML equivalent, a one-class classifier (OCC) could be defined for each disease, receiving pathologies as input. OCC are useful when data from other classes is difficult to obtain. In this case such methods would allow to define a corresponding equation for each disease, encoding its pathology particularities. The respective functions of the diseases could be updated as the disease pathology knowledge evolves, but the model used to extract the pathologies from CXR will not change in that case (unless new types of pathologies are to be found), in the same way that instruments for medical tests are rarely changed when the new knowledge of a disease is learnt.

## III. Discussion

This section discusses the limitations of inductive methods addressed before and how domain knowledge becomes essential to ease and direct its impact.

### A. On Induction

Whether to reject induction as a justification method or not is still an open debate and not the aim of this work, but at least we should agree that induction, while useful, is limited. Such limits must be taken into account, especially when the problem requires non-monotonic means because the inferences of the model are tentative and defeasible.

If we visualise the data as points in a plane; every set of finite points belongs to infinite functions or curves. The problem of induction, therefore, consists in establishing criteria that allow us to say that the finite series of data confirms only one of the functions, or less dramatically but just as problematic, that one is more confirmed than the others [59].

The confirmation of a hypothesis, or in this case the candidate model, is often considered to increase as the number of favourable test findings grows, but the increase in confirmation, produced by one new favourable instance, will generally become smaller as the number of previously established favourable instances grows [60, Ch. 4, p. 34]. Many researchers blindly rely on the dogma *the more data, the merrier* but the addition of one more favourable finding raises the hypothesis confirmation but little [61]. The confirmation of a hypothesis depends not only on the quantity of the favourable evidence available but also on its variety. To overcome this first error naïve researchers could quickly pour in other datasets into the pot, but even such well intended decision can have unexpected consequences as the model can boost the features that make the datasets different instead of their commonalities. For example, in the context of COVID-19 detection from CXR scans, Maguolo and Nanni showed how models can learn to predict features that depend on the source of the dataset rather than the relevant medical information [62]. Again, domain knowledge becomes essential to weigh the data and direct the model on which features are relevant and which are confounding. Therefore, careful data curation is crucial to prevent risk of bias in the models.

### B. On Domain Knowledge

Diagnosis is intrinsically multimodal and often requires tests of different nature to draw a conclusion, comprising multiple areas of medicine. Seeking to diagnose lung diseases with just CXR images is unnecessarily biased, and yet again, an example of how problems are often forced to fit the available data instead of fed with the data they need to become solvable. Owing to the defeasible nature of diagnosis, causal information regarding the diseases is crucial for building ML models and computer-aided diagnosis solutions.

Even though the prediction performance of ML solutions can be convincing, the lack of explicit models can make ML solutions difficult to directly relate to existing biological knowledge [63]. In this sense, Bayesian approaches may be used to embed appropriate priors from domain knowledge to better assess predictions’ confidence, which ultimately increases model robustness. Consequently, translational medicine must be bidirectional, and more effort has to be put into bringing medical knowledge into the data and model design. In the case of CXR and CT images, curation by radiologists could be immensely enlightening for the construction of better models that detect lung lesions or abnormalities. Then, pathology knowledge can pave the way to embed causal relationships between pathologies and diseases into the models.

## IV. Conclusion

This work attempts to provide some perspective to researchers of multiple areas regarding the current trend from the mainstream machine learning community to address significant challenges with careless solutions. Such solutions are oversold by meaningless KPIs unable to discern the suitability of a model for real-world clinical settings.

Several domains ranging from machine learning and epistemology to logic and pathology have been superficially tackled in this work, with a special focus on its impact on the conventional solutions developed for the automatic detection of COVID-19 from CXR images. This work focus on the automatic detection of lung diseases from CXR images as a goal, with transferability to real clinical settings as a requirement. The methodological flaws of such solutions are masked by KPIs stressing the high accuracy and precision of the models in their training and test datasets, but such solutions hide dangerous risks that may arise when transferring them into real-world clinical settings.

The epistemic issues addressed in this work concerning induction and monotonicity condition the means by which the goals are achieved and how methods are justified. The methods of such solutions were chosen by convention, disregarding the particularities of the problem at issue, and failing to consider knowledge from the domain at hand; for instance, that lung diseases are not necessarily mutually exclusive events and that diseases can share pathologies. Ignoring these facts, and ultimately ignoring domain knowledge, conditions the methods employed to achieve the aforementioned goals. The relationships between diseases and the pathologies are not in the data but do exist in reality, of which the data is merely a blurry shadow similar to the shadows of Plato’s cave. The scientific community is already responding to the many issues affecting reproducibility, interpretability and transferability of ML solutions. Efforts are being made in different fronts, establishing guidelines for datasets [2], methods [1], ethics and community challenges [64], which aim to make ML solutions more suitable for their translation into real-world clinical settings. We hope this work will continue to raise awareness on this topic and help researchers develop better solutions, and ultimately unveil knowledge.

## References

[1] M. Roberts, D. Driggs, M. Thorpe, J. Gilbey, M. Yeung, S. Ursprung, A. I. Aviles-Rivero, C. Etmann, C. McCague, L. Beer, and J. R. Weir-McCall, “Common pitfalls and recommendations for using machine learning to detect and prognosticate for COVID-19 using chest radiographs and CT scans,” Nature Mach. Intell., vol. 3, no. 3, pp. 199–217, 2021.

[2] B. G. S. Cruz, M. N. Bossa, J. Sölter, and A. D. Husch, “Public Covid-19 X-ray datasets and their impact on model bias—A systematic review of a significant problem,” medRxiv, 2021, doi: 10.1101/2021.02.15.21251775. PMC8479314 34597937

[3] D. Sculley, J. Snoek, A. B. Wiltschko, and A. Rahimi, “Winner’s curse? On pace, progress, and empirical rigor,” in Proc. 6th Int. Conf. Learn. Represent. (ICLR). Vancouver, BC, Canada: OpenReview.net, Apr./May 2018. [Online]. Available: https://openreview.net/forum?id=rJWF0Fywf

[4] L. Maier-Hein, M. Eisenmann, A. Reinke, S. Onogur, M. Stankovic, P. Scholz, T. Arbel, H. Bogunovic, A. P. Bradley, A. Carass, and C. Feldmann, “Why rankings of biomedical image analysis competitions should be interpreted with care,” Nature Commun., vol. 9, Dec. 2018, Art. no. 5217. 10.1038/s41467-018-07619-7 PMC6284017 30523263

[5] J. Morley, C. C. V. Machado, C. Burr, J. Cowls, I. Joshi, M. Taddeo, and L. Floridi, “The ethics of AI in health care: A mapping review,” Social Sci. Med., vol. 260, Sep. 2020, Art. no. 113172. 10.1016/j.socscimed.2020.113172 32702587

[6] W. N. Price, S. Gerke, and I. G. Cohen, “Potential liability for physicians using artificial intelligence,” Jama, vol. 322, no. 18, pp. 1765–1766, 2019. 31584609 10.1001/jama.2019.15064

[7] J. K. Eshraghian, “Human ownership of artificial creativity,” Nature Mach. Intell., vol. 2, no. 3, pp. 157–160, Mar. 2020.

[8] O. DeMasi, K. Kording, and B. Recht, “Meaningless comparisons lead to false optimism in medical machine learning,” PLoS ONE, vol. 12, no. 9, 2017, e0184604. 10.1371/journal.pone.0184604 PMC5614525 28949964

[9] E. J. Emanuel and R. M. Wachter, “Artificial intelligence in health care: Will the value match the hype?” Jama, vol. 321, no. 23, pp. 2281–2282, 2019. 31107500 10.1001/jama.2019.4914

[10] A. Holzinger, B. Haibe-Kains, and I. Jurisica, “Why imaging data alone is not enough: AI-based integration of imaging, omics, and clinical data,” Eur. J. Nucl. Med. Mol. Imag., vol. 46, no. 13, pp. 2722–2730, Dec. 2019. 10.1007/s00259-019-04382-9 31203421

[11] B. Russell, The Problems of Philosophy. London, U.K.: Williams and Norgate, 1912.

[12] S. Skansi, Guide to Deep Learning Basics. Cham, Switzerland: Springer, 2020.

[13] D. Hume, A Treatise Upon Human Nature. Oxford, U.K.: Oxford Univ. Press, 1739.

[14] F. Bergadano, “The problem of induction and machine learning,” in Proc. 12th Int. Joint Conf. Artif. Intell. (IJCAI), vol. 2. San Mateo, CA, USA: Morgan Kaufmann, 1991, pp. 1073–1078.

[15] P. Lakhani and B. Sundaram, “Deep learning at chest radiography: Automated classification of pulmonary tuberculosis by using convolutional neural networks,” Radiology, vol. 284, no. 2, pp. 574–582, Aug. 2017. 28436741 10.1148/radiol.2017162326

[16] O. Er, N. Yumusak, and F. Temurtas, “Chest diseases diagnosis using artificial neural networks,” Expert Syst. Appl., vol. 37, no. 12, pp. 7648–7655, Dec. 2010.

[17] O. Er, N. Yumusak, and F. Temurtas, “Diagnosis of chest diseases using artificial immune system,” Expert Syst. Appl., vol. 39, no. 2, pp. 1862–1868, Feb. 2012.

[18] S. Chen, Q. Yin, H. Shi, D. Du, S. Chang, L. Ni, H. Qiu, Z. Chen, J. Zhang, and W. Zhang, “A familial cluster, including a kidney transplant recipient, of coronavirus disease 2019 (COVID-19) in Wuhan, China,” Amer. J. Transplantation, vol. 20, no. 7, pp. 1869–1874, Jul. 2020. 10.1111/ajt.15903 PMC9800498 32243690

[19] E. Callaway and D. Cyranoski, “China coronavirus: Six questions scientists are asking,” Nature, vol. 577, no. 7791, pp. 605–608, 2020. 31992880 10.1038/d41586-020-00166-6

[20] S. J. Olsen, M.-Y. Chen, Y.-L. Liu, M. Witschi, A. Ardoin, C. Calba, P. Mathieu, V. Masserey, F. Maraglino, S. Marro, and P. Penttinen, “Early introduction of severe acute respiratory syndrome coronavirus 2 into Europe,” Emerg. Infectious Diseases, vol. 26, no. 7, p. 1567, 2020. 10.3201/eid2607.200359 PMC7323534 32197059

[21] A. Amendola, S. Bianchi, M. Gori, D. Colzani, M. Canuti, E. Borghi, M. C. Raviglione, G. V. Zuccotti, and E. Tanzi, “Evidence of SARS-CoV-2 RNA in an oropharyngeal swab specimen, Milan, Italy, early December 2019,” Emerg. Infectious Diseases, vol. 27, no. 2, pp. 648–650, Feb. 2021. 10.3201/eid2702.204632 PMC7853584 33292923

[22] (2021). Merriam-Webster. Pathology. [Online]. Available: https://www.merriam-webster.com/dictionary/pathology

[23] E. Tartaglione, C. A. Barbano, C. Berzovini, M. Calandri, and M. Grangetto, “Unveiling COVID-19 from chest X-ray with deep learning: A hurdles race with small data,” Int. J. Environ. Res. Public Health, vol. 17, no. 18, pp. 1–17, Apr. 2020. 10.3390/ijerph17186933 PMC7557723 32971995

[24] J. Paul Cohen, P. Morrison, and L. Dao, “COVID-19 image data collection,” 2020, arXiv:2003.11597. [Online]. Available: http://arxiv.org/abs/2003.11597

[25] A. Mangal, S. Kalia, H. Rajgopal, K. Rangarajan, V. Namboodiri, S. Banerjee, and C. Arora, “CovidAID: COVID-19 detection using chest X-ray,” 2020, arXiv:2004.09803. [Online]. Available: http://arxiv.org/abs/2004.09803

[26] L. Wang, Z. Q. Lin, and A. Wong, “COVID-Net: A tailored deep convolutional neural network design for detection of COVID-19 cases from chest X-ray images,” Sci. Rep., vol. 10, no. 1, Dec. 2020, Art. no. 19549. 10.1038/s41598-020-76550-z PMC7658227 33177550

[27] S. Minaee, R. Kafieh, M. Sonka, S. Yazdani, and G. Jamalipour Soufi, “Deep-COVID: Predicting COVID-19 from chest X-ray images using deep transfer learning,” Med. Image Anal., vol. 65, Oct. 2020, Art. no. 101794. 10.1016/j.media.2020.101794 PMC7372265 32781377

[28] A. I. Khan, J. L. Shah, and M. M. Bhat, “CoroNet: A deep neural network for detection and diagnosis of COVID-19 from chest X-ray images,” Comput. Methods Programs Biomed., vol. 196, Nov. 2020, Art. no. 105581. 10.1016/j.cmpb.2020.105581 PMC7274128 32534344

[29] J. Zhang, Y. Xie, G. Pang, Z. Liao, J. Verjans, W. Li, Z. Sun, J. He, Y. Li, C. Shen, and Y. Xia, “Viral pneumonia screening on chest X-ray images using confidence-aware anomaly detection,” 2020, arXiv:2003.12338. [Online]. Available: http://arxiv.org/abs/2003.12338 10.1109/TMI.2020.3040950 PMC8544953 33245693

[30] A. M. Ismael and A. Şengür, “Deep learning approaches for COVID-19 detection based on chest X-ray images,” Expert Syst. Appl., vol. 164, Feb. 2021, Art. no. 114054. 10.1016/j.eswa.2020.114054 PMC7521412 33013005

[31] M. Ahishali, A. Degerli, M. Yamac, S. Kiranyaz, M. E. H. Chowdhury, K. Hameed, T. Hamid, R. Mazhar, and M. Gabbouj, “Advance warning methodologies for COVID-19 using chest X-ray images,” IEEE Access, vol. 9, pp. 41052–41065, 2021. 10.1109/ACCESS.2021.3064927 PMC8768954 36789157

[32] B. Abraham and M. S. Nair, “Computer-aided detection of COVID-19 from X-ray images using multi-CNN and bayesnet classifier,” Biocybernetics Biomed. Eng., vol. 40, no. 4, pp. 1436–1445, Oct. 2020. 10.1016/j.bbe.2020.08.005 PMC7467028 32895587

[33] I. D. Apostolopoulos and T. A. Mpesiana, “Covid-19: Automatic detection from X-ray images utilizing transfer learning with convolutional neural networks,” Phys. Eng. Sci. Med., vol. 43, no. 2, pp. 635–640, Jun. 2020. 32524445 10.1007/s13246-020-00865-4 PMC7118364

[34] A. Abbas, M. M. Abdelsamea, and M. M. Gaber, “Classification of COVID-19 in chest X-ray images using DeTraC deep convolutional neural network,” Int. J. Speech Technol., vol. 51, no. 2, pp. 854–864, Feb. 2021. 10.1007/s10489-020-01829-7 PMC7474514 34764548

[35] S. H. Yoo, H. Geng, T. L. Chiu, S. K. Yu, D. C. Cho, J. Heo, M. S. Choi, I. H. Choi, C. Cung Van, N. V. Nhung, B. J. Min, and H. Lee, “Deep learning-based decision-tree classifier for COVID-19 diagnosis from chest X-ray imaging,” Frontiers Med., vol. 7, p. 427, Jul. 2020. 10.3389/fmed.2020.00427 PMC7371960 32760732

[36] R. M. Pereira, D. Bertolini, L. O. Teixeira, C. N. Silla, and Y. M. G. Costa, “COVID-19 identification in chest X-ray images on flat and hierarchical classification scenarios,” Comput. Methods Programs Biomed., vol. 194, Oct. 2020, Art. no. 105532. 10.1016/j.cmpb.2020.105532 PMC7207172 32446037

[37] T. Ozturk, M. Talo, E. A. Yildirim, U. B. Baloglu, O. Yildirim, and U. R. Acharya, “Automated detection of COVID-19 cases using deep neural networks with X-ray images,” Comput. Biol. Med., vol. 121, Jun. 2020, Art. no. 103792. 10.1016/j.compbiomed.2020.103792 PMC7187882 32568675

[38] S. Toraman, T. B. Alakus, and I. Turkoglu, “Convolutional capsnet: A novel artificial neural network approach to detect COVID-19 disease from X-ray images using capsule networks,” Chaos, Solitons Fractals, vol. 140, Nov. 2020, Art. no. 110122. 10.1016/j.chaos.2020.110122 PMC7357532 32834634

[39] S. Karakanis and G. Leontidis, “Lightweight deep learning models for detecting COVID-19 from chest X-ray images,” Comput. Biol. Med., vol. 130, Mar. 2021, Art. no. 104181. 10.1016/j.compbiomed.2020.104181 PMC7831681 33360271

[40] A. Narin, C. Kaya, and Z. Pamuk, “Automatic detection of coronavirus disease (COVID-19) using X-ray images and deep convolutional neural networks,” 2020, arXiv:2003.10849. [Online]. Available: http://arxiv.org/abs/2003.10849 10.1007/s10044-021-00984-y PMC8106971 33994847

[41] G. T. Mousquer, A. Peres, and M. Fiegenbaum, “Pathology of TB/COVID-19 co-infection: The phantom menace,” Tuberculosis, vol. 126, 2020, Art. no. 102020, doi: 10.1016/j.tube.2020.102020. PMC7669479 33246269

[42] D. Visca, C. Ong, S. Tiberi, R. Centis, L. D’Ambrosio, B. Chen, J. Mueller, P. Mueller, R. Duarte, M. Dalcolmo, G. Sotgiu, G. Migliori, and D. Goletti, “Tuberculosis and COVID-19 interaction: A review of biological, clinical and public health effects,” Pulmonology, vol. 27, no. 2, pp. 151–165, 2021. 33547029 10.1016/j.pulmoe.2020.12.012 PMC7825946

[43] Z. Yousaf, A. A. Khan, H. A. Chaudhary, K. Mushtaq, J. Parengal, M. Aboukamar, M. U. Khan, and M. F. H. Mohamed, “Cavitary pulmonary tuberculosis with COVID-19 coinfection,” IDCases, vol. 22, Jan. 2020, Art. no. e00973. 10.1016/j.idcr.2020.e00973 PMC7521360 33014710

[44] W. K. Funkhouser, “Pathology: The clinical description of human disease,” in Molecular Pathology: The Molecular Basis of Human Disease. Amsterdam, The Netherlands: Elsevier, Feb. 2009, pp. 197–207.

[45] B. Kelly, “The chest radiograph,” Ulster Med. J., vol. 81, no. 3, p. 143, 2012. 23620614 PMC3632825

[46] C. Delacourt, T. M. Mani, V. Bonnerot, J. de Blic, N. Sayeg, D. Lallemand, and P. Scheinmann, “Computed tomography with normal chest radiograph in tuberculous infection,” Arch. Disease Childhood, vol. 69, no. 4, pp. 430–432, Oct. 1993. 8259872 10.1136/adc.69.4.430 PMC1029549

[47] Chest Radiography: Radiographic Classification: Medical Diagnosis—NIOSHWorkplace Safety and Health Topic, CDC, Centers Disease Control Prevention, Atlanta, GA, USA, May 2011.

[48] A. Kendall and Y. Gal, “What uncertainties do we need in Bayesian deep learning for computer vision?” 2017, arXiv:1703.04977. [Online]. Available: http://arxiv.org/abs/1703.04977

[49] E. Hüllermeier and W. Waegeman, “Aleatoric and epistemic uncertainty in machine learning: An introduction to concepts and methods,” Mach. Learn., vol. 110, no. 3, pp. 457–506, Mar. 2021.

[50] C. Strasser and G. A. Antonelli, “Non-monotonic logic,” in The Stanford Encyclopedia of Philosophy, E. N. Zalta, Ed. Stanford, CA, USA: Metaphysics Research Lab, Stanford Univ., Summer 2019.

[51] K. Genin and F. Huber, “Formal Representations of Belief,” in The Stanford Encyclopedia of Philosophy, E. N. Zalta, Ed. Stanford, CA, USA: Metaphysics Research Lab, Stanford Univ., Spring 2021.

[52] L. Longo, L. Rizzo, and P. Dondio, “Examining the modelling capabilities of defeasible argumentation and non-monotonic fuzzy reasoning,” Knowl.-Based Syst., vol. 211, Jan. 2021, Art. no. 106514.

[53] J. Pearl, Probabilistic Reasoning in Intelligent Systems: Networks of Plausible Inference. San Mateo, CA, USA: Morgan Kaufmann, 1988.

[54] S. Hanks and D. McDermott, “Nonmonotonic logic and temporal projection,” Artif. Intell., vol. 33, no. 3, pp. 379–412, Nov. 1987.

[55] R. Koons, “Defeasible Reasoning,” in The Stanford Encyclopedia of Philosophy, E. N. Zalta, Ed. Stanford, CA, USA: Metaphysics Research Lab, Stanford Univ., 2017.

[56] J. Fox, D. Glasspool, and J. Bury, “Quantitative and qualitative approaches to reasoning under uncertainty in medical decision making,” in Proc. Conf. Artif. Intell. Med. Eur. Berlin, Germany: Springer, 2001, pp. 272–282, doi: 10.1007/3-540-48229-6_39.

[57] L. Longo and P. Dondio, “Defeasible reasoning and argument-based systems in medical fields: An informal overview,” in Proc. IEEE 27th Int. Symp. Comput.-Based Med. Syst., May 2014, pp. 376–381.

[58] P. Cudahy and S. V. Shenoi, “Diagnostics for pulmonary tuberculosis,” Postgraduate Med. J., vol. 92, no. 1086, pp. 187–193, 2016. 10.1136/postgradmedj-2015-133278 PMC4854647 27005271

[59] J. A. Díez and C. U. Moulines, Fundamentos de Filosofía de la Ciencia. Barcelona, Spain: Editorial Ariel, 1997.

[60] C. G. Hempel, Philosophy of Natural Science. Upper Saddle River, NJ, USA: Prentice-Hall, 1966.

[61] D. Bzdok, M. Krzywinski, and N. Altman, “Machine learning: A primer,” Nature Methods, vol. 14, no. 12, p. 1119, 2017. 29664466 10.1038/nmeth.4526 PMC5905345

[62] G. Maguolo and L. Nanni, “A critic evaluation of methods for COVID-19 automatic detection from X-ray images,” 2020, arXiv:2004.12823. [Online]. Available: http://arxiv.org/abs/2004.12823 10.1016/j.inffus.2021.04.008 PMC8086233 33967656

[63] D. Bzdok, N. Altman, and M. Krzywinski, “Points of significance: Statistics versus machine learning,” Nature Methods, vol. 15, no. 4, pp. 233–234, Apr. 2018. 30100822 10.1038/nmeth.4642 PMC6082636

[64] L. Maier-Hein, A. Reinke, M. Kozubek, A. L. Martel, T. Arbel, M. Eisenmann, A. Hanbury, P. Jannin, H. Müller, S. Onogur, J. Saez-Rodriguez, B. van Ginneken, A. Kopp-Schneider, and B. A. Landman, “BIAS: Transparent reporting of biomedical image analysis challenges,” Med. Image Anal., vol. 66, Dec. 2020, Art. no. 101796. 10.1016/j.media.2020.101796 PMC7441980 32911207

